# Associations between bio-motor ability, endocrine markers and hand-specific anthropometrics in elite female futsal players: a pilot study

**DOI:** 10.1186/s13102-022-00453-x

**Published:** 2022-04-05

**Authors:** Farid Farhani, Hamid Arazi, Mohammad Mirzaei, Hadi Nobari, Elena Mainer-Pardos, Imen Moussa Chamari, Julien S. Baker, Jorge Pérez-Gómez, Karim Chamari

**Affiliations:** 1 Department of Physical Education and Sport Sciences, Faculty of Humanities, Tarbiat Modares University, Tehran, Iran; 2 Department of Exercise Physiology, Faculty of Sport Sciences, University of Guilan, 10th km of Tehran Road- Khalij-e-Fars highway, 4199843653 Rasht, Iran; 3 Department of Physical Education and Sports, University of Granada, 18010 Granada, Spain; 4 Sports Scientist, Sepahan Football Club, 81887-78473 Isfahan, Iran; 5 Health Sciences Faculty, Universidad San Jorge, Autov A23 km 299, 50830 Villanueva de Gállego, Zaragoza, Spain; 6 Physical Education Department, College of Education, Qatar University, Doha, Qatar; 7 Centre for Health and Exercise Science Research, Department of Sport, Physical Education and Health, Hong Kong Baptist University, Kowloon Tong, Hong Kong; 8 HEME Research Group, Faculty of Sport Sciences, University of Extremadura, 10003 Cáceres, Spain; 9 Qatar Orthopaedic and Sports Medicine Hospital, FIFA Medical Centre of Excellence, Doha, Qatar

**Keywords:** Talent, Football, Digit ratio, Hormone, Skill, Power, Performance

## Abstract

**Background:**

The second-to-fourth digit ratio (2D:4D) has been calculated for individual athletes and sports, but it has not been investigated in futsal performance. Therefore, the aim of this study was to investigate any relationships between 2D:4D of the dominant and non-dominant hands and physical capacity performances, selected functional variables and hormone concentrations in elite female futsal players.

**Methods:**

Twenty-four elite female futsal players were measured for 2D:4D in the dominant (2D:4D_D_) and non-dominant (2D:4D_ND_) hand. The futsal specific performance test (FSPT), hand-grip strength (HGS) and aerobic power were also assessed. In addition, selected circulatory hormones were measured (estradiol, cortisol, growth hormone and insulin like growth factor-1). Pearson’s correlation test was used to identify correlational relationships.

**Results:**

Total test-time and performance time (total time + penalty time) for the FSPT showed a significant correlation with 2D:4D_D_ (r = 0.53, *p* = 0.005 and r = 0.55, *p* = 0.003, respectively). HGS_ND_ also displayed a significant correlation with the 2D:4D_D_ (r = 0.59, *p* = 0.002). Aerobic power and time spent running on the treadmill also showed a significant relationship with 2D:4D_ND_ (both, r = 0.54, *p* = 0.006). Cortisol showed a significant correlation with 2D:4D_D_ (− 0.58, *p* = 0.003) and 2D:4D_ND_(− 0.52, *p* = 0.008).

**Conclusions:**

The measurement of 2D:4D ratio could be an important factor in determining potential performance attributes and talent identification of elite female futsal players. Further studies are needed in this area to further examine the results presented here.

## Background

Futsal is an intermittent high-intensity game with matches composed of two halves of 20 min duration each. Effort pattern is characterized by many rest intervals (e.g., 75% of the playing actions last 1–18 s), whereas more than 83% of the resting intervals are of 1–15 s duration [1]. In addition, skills such as dribbling, long pass, short pass, ball control, rotation, and shoot are performed at high speeds, demonstrating the importance of power in futsal [2]. Although factors such as strength, endurance, power and balance are important in futsal, the evaluation of a futsal player should also depend on her/his specific futsal skills [3]. In this regard, Farhani et al. [2], recently developed a futsal specific tests, called the futsal special performance test (FSPT), comprising most of the physical skills performed during match conditions.

The increasing popularity of female team sports has resulted in increased interest concerning female physiology and adaptive responses to exercise [4–7]. The regulation and production of cortisol, estradiol, growth hormone (GH) and insulin-like growth factor-1 (IGF-1) depends on physical exercise [8]. Cortisol regulates most catabolic adaptations to exercise training, and is used as an indicator of physical stress [9]. Increased vasodilation of coronary arteries and stimulation of insulin production may occur due to increase plasma estradiol levels [10]. Finally, when GH production is suppressed, performance and exercise tolerance are reduced, whereas endurance training increases circulating IGF-1 levels [11]. Despite increased research, hormonal responses to exercise are scarce in female futsal players [12].

There is evidence to suggest a significant association between functional, psychological, and even sexual components and finger length ratios (2D:4D) in runners and soccer players [13].

Ribeiro et al. [14] have previously suggested that 2D:4D finger formation may be unrelated to the function of prenatal sex hormones and the production of sex steroids. Based on the research on 2D:4D ratio of Klimek et al. [15], the ratio in male embryos is significantly lower than female embryos, and gestational age is not related to the mean value. However, 2D:4D ratios were significantly different in 2-year-old children [14]. However, low concentrations of estrogen and high testosterone in the mother's blood may be associated with a low 2D:4D ratio [16]. The negative correlation between testosterone and 2D:4D ratio seen in men during pregnancy is likely to change during growth and puberty [14]. Also, it has been reported that females with high levels of sex hormones have a low 2D:4D ratio [17].

One of the outcomes associated with exposure to testosterone in the uterus is a masculinized 2D:4D hand dominance, and researchers have examined the relationship between the dominant hand and the 2D:4D ratio. However, the data showed an inverse, null, and direct relationship with left-hand dominance [14]. Despite the increase in the number of left-handed individuals in populations exposed to diethylstilbestrol, it has been hypothesized that left-handed dominance is associated with high uterine estrogen exposure. In contrast, a study found an association between 2D:4D lower right ratio and left-handed writing preference, which confirms high testosterone exposure during pregnancy and left-hand dominance [18]. Exposure to intrauterine sex hormones on selective sexual adaptations is thought to play a key role in female intrasexual competition. Also, several studies have examined the relationship between 2D:4D ratios and physical fitness parameters and found a significant correlation in this regard with the variables of performance, strength, power and endurance of male basketball players [19].

Various methods have been used by researchers to measure the 2D:4D ratios. These include scanned images of the palm [20], photocopies [21], or hand outline drawings. Radiographic methods have also been used [22]. However, methodological challenges have also been emphasized by Vehmas et al. [23], who did not find any relationships between 2D:4D measured from radiographs and body mass index, fat percentage, psychological, occupational or fertility variables in females. Radiographs and nuclear images have side effects and may endanger health (due to radiation). A scan or X-ray is used if there are clear medical reasons. Also, photocopiers may not be able to report the actual size of the finger length due to ink dispersion and skin challenges [24]. Finally, Visnapuu and Jürimäe [25] observed that the outlined 2D:4D method is the most convenient method due to its simplicity, lower costs, minimal use of equipment, accessibility of subjects and importantly, good reliability.

As mentioned previously, 2D:4D ratio has been measured in many individual athletes and sports [13], and has proven to be important for talent identification in females, but the ratio has not been investigated with regard to futsal performance. Moreover, futsal has become increasingly popular among females worldwide [26], nevertheless scientific knowledge about the physiological responses to exercise in futsal populations is limited. Therefore, the aim of this study, was to investigate possible relationships among different dominant and non-dominant-hand finger-length ratios and FSPT, functional factors and hormone concentrations in a sample of elite female futsal players. We assumed that there would be significant correlations between the above variables.

## Methods

### Participants

The subjects of this study were 24 female futsal players who had participated for at least 5 years in Iran’s National Premier League. The present research was conducted during 8-week pre-season period. Participant characteristics were as follows: age 23.9 ± 3.7 years, body height 163.4 ± 2.6 cm, body mass 58.4 ± 7.3 kg and body mass index (BMI) 21.9 ± 2.7 kg/m^2^. Stature and mass were recorded using a calibrated stadiometer and weighing scale respectively. BMI was calculated using individual subject weight in kilograms and their height in metres squared. All anthropometric measures were recorded in session 1. All participants were healthy without any orthopaedic, neuromuscular disorders or cardiovascular diseases. They signed an informed consent form to participate before starting the study. The study was approved by the University of Guilan review board and was performed in accordance with the ethical standards laid down in the latest version of Declaration of Helsinki.

### Protocol

The research protocol was implemented in June 2019 for four consecutive days. Players participated in 4 sessions: (1) familiarization session; (2) blood sampling and 2D:4D measurement trial 1 was performed in the lab between 8 to 11 am, afterward, FSPT was measured in the Futsal Hall between 15 to 19 pm; (3) hand-grip strength trial and 2D:4D measurement trial 2 were performed in the lab between 9 to 11 am, and (4) hand-grip strength trial 2 and the Bruce test were performed in the morning and evening, respectively. The inclusion of each participant in the present study depended on individual menstrual cycle phases. Also, a Visual Analogue Scale (VAS) standard questionnaire was used to accurately determine menstrual disorders [27]. After determining menstruation (all participants had regular menstruations), each participant was asked to attend the lab at the specified time and day (follicular phase). Blood samples were taken during the early follicular phase (5–7 days) of the menstrual cycle [28].

### 2D:4D measurement

**Fig. 1:**
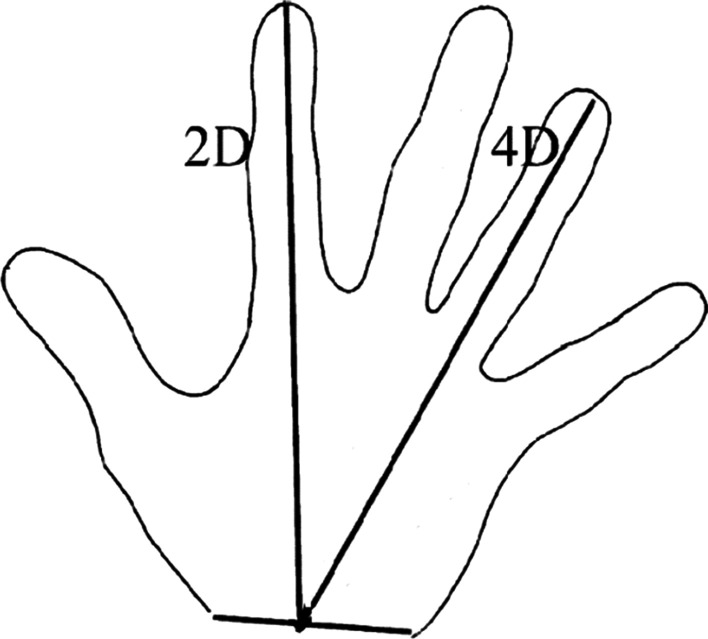
Measurement of length parameters of the hand. Measurement of second-to-fourth digit

For the measurement of second-to-fourth digit ratio, the participants were instructed to place their hand on a desk, spread and stretch out the hand on to a blank paper sheet placed on a desk and the profile of the hand was then carefully drawn. The original method of Visnapuu and Jürimäe was used for the measurement of second-to-fourth digit ratio. Finger length (see Fig. 1) was measured between the wrist joint (distal wrist crease) and the tip of the fingers: length from the wrist joint to the tip of the index fingers (2D) and length from the wrist joint to the tip of the ring finger (4D) [12]. This method has been shown to be highly reliable [25]. We defined 2D:4D_D_ (as the dominant hand) and 2D:4D_ND_ (as the non- dominant hand). We also investigated the reliably of this method within our experimental group. We replicated drawings of multiple hands, which were copied twice. We then presented them to an experimenter, in a random fashion containing only a number (anonymized drawings).

The blinded experimenter then had to measure the 2D:4D for each drawing. Pearson's correlation test showed a strong correlation between the drawn image results (dominant hand = 0.98 and non-dominant = 0.97).

### Futsal special performance test

The FSPT assesses the performance and skill of futsal players. This test consists of nine steps including running with the ball, dribbling, turning, long and short passes, receiving a pass, performing a wall pass, shooting and sprinting without the ball. Time related performances recorded were total time and penalty time. The penalty time which included errors when performing the test was calculated as follows: (1) ball hitting cone: 2 s, (2) wrong pass: 2 s, (3) no goal and completely missing the goal framework: 2 s, (4) ball hitting goal framework: 1 s. The performance time was calculated as the penalty time added to the total time. The positive outcome of the test relates to the lower total time and performance time, indicating individual player's skill and power. The FSPT was performed in accordance with the recommendations of Farhani et al. [2].

### Hand-grip strength

The participants performed a hand-grip strength (HGS) test using a digital dynamometer (Takei Kiki Kogyo grip dynamometer, Japan). Three measurements of maximum strength on two consecutive days were obtained for both hands (Dominant and non-Dominant), and mean values recorded. This method has been shown to be highly reliable [29]. In addition, we were also able to demonstrate the reliably of this method. Pearson's correlation test showed a strong correlation between the test and re-test results for HGS (Dominant hand = 0.98 and non-Dominant hand = 0.97).

### Aerobic power

To estimate aerobic power by maximal oxygen consumption values (VO_2max_), the Bruce treadmill protocol was used [30]. Briefly, participants ran on a treadmill completing an exhaustive running test. Following a warm up for 10 min at a comfortable speed; gradient and speed were increased gradually from 2.0 to 4.5 mph at a 0% grade that elicited a heart rate response between 50 to 60% of the maximum heart rate. Thereafter, participants performed an exhaustive run starting at 1.7 mph/h and 10% slope for 3 min. The treadmill speed and incline were increased by 0.8 mph/h and 2% every 3 min until the participants were exhausted (see Eq. 1). Full protocols and gas collection methods and detailed methodologies have been described in detail previously [30].

1 $${\text{Aerobic}}\;{\text{power}} = 4.38 \times {\text{T}} - 3.9$$

The Bruce protocol calculation for female aerobic power (ml/kg/min). T = Total time on the treadmill measured as a fraction of a minute (a test time of 9 min 30 s would be written as T = 9.5).

### Blood sampling

A 5 ml blood sample was taken from the antecubital vein and collected in ethylenediaminetetraacetic (EDTA) tubes. The participants were fasted (for the last ~ 12 h) and the samples were collected between 8 and 9 am. The blood samples were taken during the early follicular phase (5–7 days) of the menstrual cycle of the participants [12]. Blood samples were centrifuged (-4C, 3000 rpm) for 10 min to isolate the serum and stored at − 20C. Serum samples were analysed for estradiol, cortisol, GH and IGF-1 by enzyme immunoassay (EIA) (kit IBL, MD58011, Hamburg, Germany). The inter- and intra- assay variance were < 10% [31].

### Statistical analysis

Statistical analyses were performed using SPSS for MAC (Version 24.0; SPSS Inc, Chicago, IL). Data are presented as mean ± standard deviations (SD). The normality of data for all the studied variables was confirmed using the Shapiro–Wilk test. Pearson’s correlation parametric test was used to investigate relationships between variables, and stepwise multiple regression analysis models were also used. In addition, *R*^*2*^*, β* and 95% confidence intervals (*95% CI*) were calculated for all significant correlations. Statistical significance was inferred from *p* < 0.05.

## Results

The 2D:4D of the dominant and non-dominant hand, along with futsal specific performance, functional factors and hormones of the participants are shown in Table 1. The total time and performance time variables showed a significant relationship with the 2D:4D_D_(Table 2). HGS_ND_ also showed a significant correlation in the 2D:4D_D._ Aerobic power and time on the treadmill showed a significant relationship with the 2D:4D_ND_ (Table 2). Cortisol showed a significant correlation with both 2D:4D. Finally, BMI and body mass showed a significant correlation with 2D:4D_ND_ (Table 2).

**Table 1:** Mean ± standard deviation **(**SD) of measured variables in futsal players

Variable	Mean ± SD	Ccoefficient of variation
2D:4D_D_	1.014 ± 0.06	0.049
2D:4D_n-D_	0.989 ± 0.05	0.050
Total time (s)	36.37 ± 5.04	0.138
Penalty time (s)	2.863 ± 2.71	0.946
Performance time (s)	39.28 ± 6.23	0.158
HGS_D_ (kg)	29.08 ± 4.40	0.151
HGS_n-D_ (kg)	28.33 ± 4.17	0.147
Aerobic power (ml/kg^−1^/min^−1^)	41.46 ± 5.64	0.136
Time on the treadmill (min)	13.98 ± 4.74	0.338
Estradiol (pmol/l)	97.32 ± 48.39	0.497
Cortisol (nmol/l)	415.8 ± 99.61	0.239
GH (ng/ml)	11.52 ± 2.24	0.194
IGF-1 (ng/ml)	85.00 ± 20.20	0.237

2D:4D_D_: Second digit to fourth Digit ratio of Dominant Hand, 2D:4D_n-D_: Second digit to fourth Digit ratio of non-Dominant Hand, HGS: hand-grip strength, GH: Growth hormone, IGF-1: Insulin-like growth factor-1

**Table 2:** Correlations among variables

	Correlation	2D:4D_D_	2D:4D_ND_
Total time (min)	*r*	0.53	0.03
	*p*	0.005**	0.87
Penalty time (s)	*r*	0.30	0.02
	*p*	0.13	0.92
Performance time (s)	*r*	0.55	0.01
	*p*	0.003**	0.93
HGS_D_ (kg)	*r*	0.29	0.22
	*p*	0.10	0.28
HGS_ND_ (kg)	*r*	0.59	0.28
	*p*	0.002**	0.18
Aerobic power (ml/kg^−1^/min^−1^)	*r*	0.04	0.63
	*p*	0.82	0.001**
Time on the treadmill (s)	*r*	0.04	0.63
	*p*	0.821	0.001**
Estradiol (pmol/l)	*r*	0.34	0.01
	*p*	0.09	0.94
Cortisol (nmol/l)			
	*r*	− 0.58	− 0.52
	*p*	0.003**	0.008**
GH (ng/mL)	*r*	0.05	0.10
	*p*	0.80	0.45
IGF-1 (ng/mL)	*r*	0.04	0.15
	*p*	0.83	0.45
Age (yrs)	*r*	0.12	0.07
	*p*	0.57	0.72
Body mass (kg)	*r*	0.08	0.52
	*p*	0.70	0.008**
Height (cm)	*r*	0.08	0.19
	*p*	0.69	0.37
BMI (kg/m^2^)	*r*	0.01	0.40
	*p*	0.93	0.04**

^*^P < 0.05, **P < 0.01, 2D:4D_D_: Second digit to fourth Digit ratio of Dominant Hand, 2D:4D_n-D_: Second digit to fourth Digit ratio of non-Dominant Hand, HGS_D_: hand-grip strength of Dominant Hand.; HGS_ND_: hand-grip strength of non-Dominant Hand. GH: Growth hormone, IGF-1: Insulin-like growth factor-1; BMI: Body mass index;

Stepwise regression analysis revealed that total time, performance time, HGS_ND_, VO_2max,_ time on the treadmill_*,*_ cortisol and body mass were all correlated to 2D:4D variables (Table 3).

**Table 3:** Stepwise multiple regression analysis models

Variable	Ratios	R^2^	F-value	*p *Value	β	95% CI
Total time	2D:4D_D_	0.283	9.491	0.005 **	0.532	− 0.048 to − 0.009
Performance time	2D:4D_D_	0.283	10.850	0.002 **	0.558	− 0.038 to − 0.009
HGS_n-D_	2D:4D_D_	0.407	15.075	0.001 **	0.638	− 0.016 to − 0.005
Aerobic power	2D:4D_n-D_	0.401	14.732	0.001 **	0.633	0.024 to 0.081
Time on the treadmill	2D:4D_n-D_	0.401	14.732	0.001 **	0.633	0.796 to 2.668
Cortisol	2D:4D_D_	0.336	11.151	0.003 **	0.580	− 0.001 to ≤ 0.0001
	2D:4D_n-D_	0.280	8.568	0.008 **	0.529	≤ 0.0001 to ≤ 0.0001
Body mass	2D:4D_n-D_	0.277	8.431	0.008 **	0.526	0.001 to 0.006
BMI	2D:4D_n-D_	0.215	6.035	0.022 *	0.464	0.001 to 0.016

^*^P < 0.05, **P < 0.01, 2D:4D_D_: Second digit to fourth Digit ratio of Dominant Hand, 2D:4D_n-D_: Second digit to fourth Digit ratio of non-Dominant Hand; HGSn-D: hand-grip strength of non-Dominant Hand. BMI: Body mass index

## Discussion

The aims of this study were to analyze the associations between bio-motor ability, endocrine markers and anthropometrics in elite female futsal players. The main findings of this study showed that the FSPT was significantly correlated with 2D:4D finger-length ratios in elite futsal female players. The traditionally used 2D:4D ratio was also correlated with functional factors HGS, aerobic power and cortisol.

There was a strong correlation between the FSPT and 2D:4D_D_. Farhani et al. [2], referred to the FSPT test as a novel futsal specific performance test, which was highly correlated with the Wingate anaerobic test (r = 0.5 to 0.91). In addition, Amani-Shalamzari et al. [31], following three weeks of small sided games and blood flow restriction in male futsal players, reported a decrease in total time to complete the tasks which resulted in increased performance. Hull et al. [16], studied the relationship between 2D:4D ratio and sport performance in female rowers, the conclusion was that females with smaller 2D:4D rowed substantially faster than females with larger 2D:4D. They suggested that the 2D:4D ratio would possibly be linked to underlying characteristics which have been optimized over time resulting in better rowing performance. Frick et al. [32], reported, that there were significant differences between male basketball competitive standards for the 2D:4D_Lelt_, but not for the 2D:4D_Right_. They concluded that the 2D:4D can discriminate between basketballers competing at different levels of play [32]. However, care must be taken when interpreting these results. Given that a masculinized 2D:4D_D_ is also a marker of in utero testosterone exposure [14], it is best to place the dominant hand against the non-dominant hand rather than the right hand against the left hand to better understand the correlation between physical performance and 2D:4D.

The mechanisms underlying the so-called crossover effect—when a unilateral intervention results in bilateral changes—are still unclear, but clinical applications related to lower extremity strengthening, fatigue, and stretching are already being explored by sports science specialists [33]. HGS_ND_ was significantly correlated with 2D:4D_D_ (r = 0.59). This finding may be related to a cross over effect [33]. In support of this, Shen et al. [34], showed, that 2D:4D in both hands were significantly and negatively correlated with HGS in females but not in males. Lu et al. [35], reported the same result in Chinese populations of Ningxia Hui ethnicity, however, Hone et al. [36], found a controversial significant correlation between HGS and 2D:4D in male, but not in female participants. The reason for these inconsistencies in general results (results expressed according to right/left laterality and not hand dominance) are probably because in these studies only the right hand was used to measure HGS.

In the present study, aerobic power and time recorded on the treadmill were significantly correlated with 2D:4D_ND_, but there was no significant correlation recorded for 2D:4D_D_. Manning et al. [37] showed a high correlation between endurance running and 2D:4D in both hands [37]. Also, Hill et al. [38], reported that 2D:4D was associated with the VO_2max_ of adolescent male players of different sports. They suggested that 2D:4D is linked to performance in some sports because it is a proxy of high sensitivity to prenatal and maybe also circulating testosterone and VO_2max_ [38]. Holzapfel et al. showed that there were no significant correlations between 2D:4D and aerobic power [39]_*.*_ The surprising aspect of our result was the significant inverse correlation observed for some variables (between HGS_ND_ with 2D:4D_D_ and aerobic power with 2D:4D_ND_). Previously, Beaton et al. reported a similar finding but did not provide any clear biological reason to explain the outcome [40]. Nonetheless, it is necessary to be cautious and it is possible that there is a relationship between 2D:4D digit ratio and other unknown factors that mediate a relationship between digit ratio and hormone concentrations or functional factors.

There were significant correlations between cortisol with 2D:4D_D_ (r = − 0.58) and 2D:4D_ND_ (r = − 0.52). Cortisol is a steroid and catabolic hormone, and it has the opposite effect of testosterone and GH [41]. Crewther et al. found a significant relationship between resting C and 2D:4D [42]. However, Beaton et al. findings were not consistent with our results, nor were there any significant relationships between concentrations of resting cortisol and either hand preference or asymmetry when performing manual skills [40]. Therefore, although we showed that cortisol had a significant relationship with measures of the hand (Dominant and non-Dominant) in female futsal players, further research is needed to provide a further understanding of the mechanisms involved. Contrary to cortisol, estradiol showed no significant relationship with the 2D:4D. Estradiol is a sex hormone that is involved in the development of female sexual organs and secondary sexual characteristics. In females, the amount of estradiol changes in a periodic period, with the highest amount observed prior to ovulation. Therefore, estradiol concentrations vary considerably over the course of a month depending on the phases of the menstrual cycle (e.g. follicular stage or luteal stage), and this finding is subject to research limitations in this area [43]. In this context, we recorded the stages of the menstrual cycle using the VAS questionnaire [27]. Hence, quantifying estradiol concentrations is methodologically important in females, and if they are involved in an elite sport level, identification of concentrations will be become more important (due to effects of exercise intensity and menstrual cycle interactions) [44].

This study has some limitations that should be recognized. These include the absence of a control group in addition to the potential of spurious correlation between variables, ensuring future investigations in the field. Furthermore, female futsal players have particular characteristics and for this reason, our results cannot be directly extrapolated to other sports and genders. Finally, no playing position differences were considered. Futsal performance has a variety of physiological demands, such as agility, endurance and muscle coordination, but in the present study only FSPT, HGS and VO_2 max_ relationships with 2D:4D were investigated, which some parameters correlated with the dominant or non-dominant 2D:4D. Therefore, it is recommended to investigate further the performance variables of power, agility and other specific Futsal tests to investigate further any correlations with 2D:4D ratio.

## Conclusions

In conclusion, the present pilot study showed that in female futsal players, the FSPT was correlated with 2D:4D. In addition, the ratio was also correlated with important variables in female futsal players, i.e. endurance and strength. Therefore, the measurement of the dominant hand 2D:4D fingers ratio seems to be a factor related to performance in futsal players. Further studies should test the present findings, and investigate the potential of the 2D:4D ratio in player talent identification programs.

## Data Availability

The datasets generated during and analyzed during the current study are available from the corresponding author on reasonable request.

## Abbreviations

2D:4D: The second-to-fourth digit ratio
2D:4D_D_: 2D:4D in the dominant hand
2D:4D_ND_: 2D:4D in the non-dominant hand
FSPT: The futsal specific performance test
HGS: Hand-grip strength
BMI: Body mass index
GH: Growth hormone
IGF-1: Insulin-like growth factor-1
VO_2max_: Maximal consumption oxygen
EDTA: Ethylenediaminetetraacetic
EIA: Enzyme immunoassay
SD: Standard deviation
CI: Confidence intervals
